# A pathway from low socioeconomic status to dementia in Japan: results from the Toyama dementia survey

**DOI:** 10.1186/s12877-018-0791-6

**Published:** 2018-04-27

**Authors:** Nobue Nakahori, Michikazu Sekine, Masaaki Yamada, Takashi Tatsuse, Hideki Kido, Michio Suzuki

**Affiliations:** 1 Faculty of Nursing Science, Tsuruga Nursing University, 78-2-1 Kizaki, Tsuruga, Fukui, 914-0814 Japan; 2 Department of Epidemiology and Health Policy, Graduate School of Medicine and Pharmaceutical Sciences, University of Toyama, 2630 Sugitani, Toyama, Toyama 930-0194 Japan; 3 Kiseikai, Kido Clinic, 244 Honoki, Imizu, Toyama, 934-0053 Japan; 4 Department of Neuropsychiatry, Graduate School of Medicine and Pharmaceutical Sciences, University of Toyama, 2630 Sugitani, Toyama, Toyama 930-0194 Japan

**Keywords:** Dementia, Socioeconomic status, Educational attainment, Occupations

## Abstract

**Background:**

The association between low socioeconomic status (SES) and dementia is reportedly mediated by lifestyle-related diseases (i.e., diabetes) in European countries and the United States; however, in Japan, the link between low SES and dementia has not been investigated. This study evaluated the possibility of a mediating role of lifestyle-related diseases in the relationship between low SES and dementia in Japan.

**Methods:**

A retrospective case-control study design, with data from the Toyama Dementia Survey, Japan, was used. Individuals aged ≥65 years (institutionalized and noninstitutionalized) living in Toyama prefecture were randomly selected, with a sampling rate of 0.5%. Of them, 1303 agreed to participate (response rate 84.8%). Overall, 137 cases of dementia and 1039 unimpaired controls were identified. Structured interviews with participants and family members or proxies were conducted, if necessary. Participants’ history of medically diagnosed disease, lifestyle factors (i.e., smoking and alcohol drinking habits), and SES (educational attainment and occupational history) were assessed. The possibility of low SES being a risk factor for dementia via lifestyle-related diseases was investigated using the Sobel test.

**Results:**

The odds ratio (OR) for dementia was higher for participants with low educational attainment (6 years or less) than for highly educated participants [age- and sex-adjusted OR 3.27; 95% confidence interval (CI) 1.84–5.81]; it was also higher for participants with a blue-collar job history than a white-collar job history (age- and sex-adjusted OR 1.26; 95% CI 0.80–1.98). After adjustment for employment history, the OR for dementia for participants with low educational attainment was 3.23–3.56. Former habitual alcohol consumption and a medical history of diabetes, Parkinson’s disease, stroke, and angina pectoris/cardiovascular disease were found to increase the risk of dementia. Educational attainment was not associated with alcohol consumption, smoking, diabetes, Parkinson’s disease, stroke, or cardiovascular disease. Occupational history was associated with diabetes and stroke. The role of diabetes in low educational attainment and dementia was found to be extremely limited.

**Conclusions:**

In Japan, lifestyle-related diseases play a minimal role as mediators between low SES and dementia.

## Background

In Japan, the prevalence of dementia is increasing as the aging population increases. The Japanese Dementia Strategy estimated the total number of individuals with dementia to be 4.62 million in 2012 and estimated that this number will increase to 7.0 million in 2025 (i.e., approximately one in five senior citizens) [1]. Following the introduction of public long-term care insurance in 2000, a range of care services have been provided to individuals with dementia, and the monetary amount of care payments has increased and is continuing to increase annually [2]. The cost of dementia in Japan is approximately 1.45 billion JPY (14.2 million USD) annually [3]. In 2012, approximately 101,000 people left their jobs to care for a family member with dementia [4]. Approximately 50% of these individuals were aged 40–59 years [5]. If this trend continues, it could have negative impacts on the Japanese economy, exacerbating existing labor-shortage issues.

In this context, it is important to support the families of individuals with dementia and to protect individuals against the development of this disease. Protecting against dementia requires the identification of predictive factors. Established dementia risk factors include genetic precursors, high blood pressure, diabetes, hyperlipidemia, and smoking [6]. Establishing a habit of regular exercise, eating a healthy diet, engaging in recreational activities, and maintaining an active social life reduce the risk of dementia [6]. Because some dementia risk factors are acquired and modifiable (e.g., cardiovascular and lifestyle-related diseases), diet and exercise interventions may reduce the risk of the disease. However, some unmodifiable factors appear to be associated with dementia as well [7]. Research conducted in Europe suggests that there is a relationship between dementia and socioeconomic factors (e.g., low levels of education and a blue-collar work history) [8–15]; however, there has been little research like this conducted in Japan [16–18]. In Europe, it has also been established that low socioeconomic status (SES) is associated with less healthy lifestyles and poorer general health, which suggests the possibility of a pathway from low SES to dementia via lifestyle factors and health inequalities. Such SES inequalities in health have not often been observed in Japan, however. Thus, it is unknown whether lifestyle-related diseases play a mediating role in the association of low SES with dementia in Japan.

We investigated whether SES is a potential risk factor for the development of dementia and how lifestyle-related diseases mediate between SES and dementia in the Toyama Dementia Survey.

## Methods

This was a retrospective case-control study.

### Participants

**Fig. 1:**
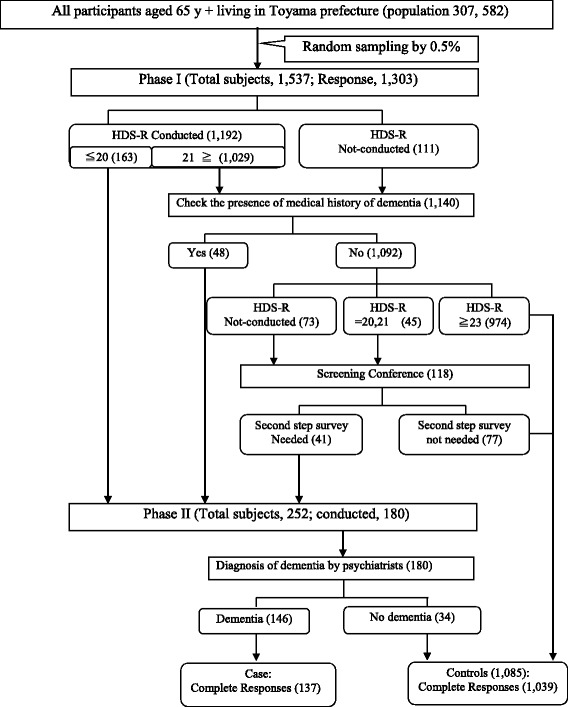
Flow Chart of the Toyama Dementia Survey

Participants were 1537 individuals, all residents of Toyama prefecture and aged ≥65 years, selected from the Basic Resident Register, on October 1, 2013. They were selected from the personal number of the basic resident register, which was randomly generated by the computer. First, the public health nurses phoned those individuals and explained the purpose of the research, and once consent was obtained, they visited the participants at a later date. Both institutionalized and noninstitutionalized individuals were recruited (total population = 307,582; sampling rate: 0.5%). Of those individuals, 1303 agreed to participate (response rate: 84.8%). A two-phase study design was used in accordance with the previous research [19]. During phase I, public health nurses visited participants’ homes or institutions and conducted face-to-face interviews using a questionnaire that included the Hasegawa Dementia Scale-Revised (HDS-R) [20]. Family members and institution staff supported this process as necessary; data were acquired from them when participants were unable to answer. The HDS-R is widely used to screen for dementia in Japan and has acceptable validity, with a sensitivity and specificity (cutoff score = 20/21) of 0.90 and 0.82, respectively. Scores on the HDS-R and the Mini-Mental State Examination are strongly correlated [21]. The correlation coefficient of the HDS-R to MMSE was reported to be as high as 0.94 [22]. Participants who scored < 20 on the HDS-R or reported a history of dementia continued into phase II of the study. Borderline participants were judged comprehensively at the screening conference and were also included in phase II. During phase II, psychiatrists and public health nurses visited the participants’ homes or institutions and diagnosed dementia according to *International Classification of Diseases*, *Tenth Revision* (*ICD-10*) criteria [23]. The ICD criteria are used worldwide and have been in use since around 1985, when the Toyama Dementia Survey began. The concordance between *ICD-10* and the *Diagnostic and Statistical Manual of Mental Disorders*, *Third Edition* (*DSM-Ш*) for the diagnosis of vascular dementia is 100% [24]. Phase I survey was conducted between June and August 2014; the phase I screening conference was conducted in September 2014, whereas the phase II survey was conducted between October and December 2014. For a control group, we used participants who scored > 23 on the HDS-R, participants who were judged inappropriate for inclusion in phase II, and participants who were not diagnosed with dementia during phase II. Finally, complete responses from 1176 participants (137 dementia cases and 1039 controls) were analyzed (Fig.1).

Participation in the Toyama Dementia Study was voluntary. All participants or their family members provided written informed consent prior to participation. The University of Toyama ethics committee approved the study protocol.

### Demographic factors and SES

Participants were examined in their sex, age, educational attainment, occupation, lifestyle factors, and medical history. For SES assessment, educational attainment and occupation of participants were examined. Educational attainment was stratified as follows: ≤6 years (elementary school), 7–9 years (higher than elementary school or through junior high school), and ≥ 10 years (high school, junior high school under the old system, girls’ high school under the old system, high school under the old system, technical school, or university).

In accordance with the Japan Standard Occupational Classification [25], occupations were classified as white collar, blue collar, both, or other. White-collar employment was defined as administrative or managerial jobs, specialist professional jobs, and clerical, sales, or service jobs. Blue-collar employment was defined as jobs in security, agriculture, forestry or fisheries, manufacturing, transport or machine operations, construction or mining, and in carrying, cleaning, packaging, and the like. “Both” was used to refer to those who had held both white- and blue-collar jobs and “other” to those who had held other types of employment (e.g., housewifery).

### Lifestyle factors

Alcohol consumption and smoking habits were examined. For alcohol consumption, participants were classified as current drinkers, former drinkers, or nondrinkers, with current drinkers being those who consumed alcohol daily, occasionally, or within a year; former drinkers being those who had ceased alcohol consumption one or more years previously; and nondrinkers being those who had never habitually consumed alcohol. Similarly, for smoking, participants were classified as current smokers, former smokers, or nonsmokers, with current smokers being those who smoked daily or within a year; former smokers being those who had stopped smoking one or more years previously; and nonsmokers being those who had never habitually smoked.

### Medical history

The medical history of participants was assessed in accordance with *ICD-10* criteria [23]. The conditions of interest for this study were diabetes, hyperlipidemia, depression, schizophrenia, Parkinson’s disease, eye disease, ear disease, hypertension, stroke, angina pectoris/cardiovascular disease, asthma, chronic obstructive pulmonary disease, any digestive system-related disease, dental disease/oral disease, rheumatoid arthritis, arthrosis, osteoporosis, kidney disease, prostatic disease, any gynecological-related disease, bone fracture, injury/scald (except for bone fracture), anemia/blood disease, and malignant neoplasm.

### Statistical analysis

Chi-square tests were used to determine whether socioeconomic, lifestyle, or medical history variables differed between participants with and without dementia. A logistic regression analysis was used to test whether SES was associated with dementia, with dementia as the dependent variable and sex, age, lifestyle factors, and medical history as the independent variables. We created several multivariate models to test whether lifestyle factors or medical history explained any observed associations between socioeconomic factors and dementia [26]. First, we calculated the age- and sex-adjusted odds ratio (OR) for dementia based on educational attainment, occupation, and socioeconomic factors. Lifestyle variables were subsequently added to the model. Then, medical history variables with *p* values < 0.25 (by chi-square testing) were added [26]. Moreover, we conducted a logistic regression analysis using the variables of lifestyle and medical history that were associated with dementia in the former analysis as dependent variables and SES, sex, and age as independent variables. We examined the fitness of the logistic regression model using the Hosmer-Lemeshow test. We investigated the possibility of a correlation between low SES and dementia via lifestyle-related diseases using the Sobel test. We used SPSS, version 23, for all analyses. ORs and 95% confidence intervals (CIs) were calculated for the logistic regression analysis.

## Results

The demographic characteristics of the participants are shown in Table 1. The proportion of participants with dementia differed significantly depending on age, educational attainment, occupation, alcohol consumption, history of diabetes, Parkinson’s disease, stroke, angina pectoris/cardiovascular disease, and bone fracture.

**Table 1:** Participant Characteristics

			Presence of dementia				
			Dementia (*n* = 137)		No dementia (*n* = 1039)		*P*
			*n*	%	*n*	%	
Attribution	Age	65–74	17	12.4	552	53.1	< 0.001
		75–84	52	38.0	371	35.7	
		≥85	68	49.6	116	11.2	
	Sex	Male	57	41.6	453	43.6	0.714
		Female	80	58.4	586	56.4	
Educational attainment	Years of education	≤6 years	36	26.3	42	4.0	< 0.001
		7–9 years	42	30.7	388	37.3	
		≥10 years	59	43.1	609	58.6	
Occupations	Occupations held	White collar	41	29.9	427	41.1	0.013
		Blue collar	58	42.3	375	36.1	
		Both	22	16.1	174	16.7	
		Other	16	11.7	63	6.1	
Lifestyle	Alcohol consumption habits	Current drinker	21	15.3	367	35.3	< 0.001
		Former drinker	33	24.1	80	7.7	
		Nondrinker	83	60.6	592	57.0	
	Smoking habits	Current smoker	6	4.4	102	9.8	0.050
		Former smoker	33	24.1	287	27.6	
		Nonsmoker	98	71.5	650	62.6	
Medical history	Diabetes	Yes	35	25.5	155	14.9	0.003
	Hyperlipidemia	Yes	19	13.9	188	18.1	0.282
	Depression	Yes	3	2.2	18	1.7	0.727
	Schizophrenia	Yes	0	0.0	4	0.4	1.000
	Parkinson’s disease	Yes	7	5.1	10	1.0	0.002
	Eye disease	Yes	30	21.9	222	21.4	0.912
	Ear disease	Yes	4	2.9	44	4.2	0.646
	Hypertension	Yes	59	43.1	489	47.1	0.413
	Stroke	Yes	26	19.0	77	7.4	< 0.001
	Angina pectoris/ cardiovascular disease	Yes	25	18.2	81	7.8	< 0.001
	Asthma	Yes	2	1.5	25	2.4	0.761
	Chronic obstructive pulmonary disease	Yes	1	0.7	9	0.9	1.000
	Digestive system disease	Yes	17	12.4	119	11.5	0.776
	Dental disease/oral disease	Yes	10	7.3	131	12.6	0.092
	Rheumatoid arthritis	Yes	2	1.5	29	2.8	0.569
	Arthrosis	Yes	14	10.2	132	12.7	0.491
	Osteoporosis	Yes	14	10.2	108	10.4	1.000
	Kidney disease	Yes	4	2.9	43	4.1	0.645
	Prostatic disease	Yes	9	6.6	66	6.4	0.854
	Gynecologic disease	Yes	6	4.4	51	4.9	1.000
	Bone fracture	Yes	40	29.2	144	13.9	< 0.001
	Injury/scald except for bone fracture	Yes	11	8.0	62	6.0	0.346
	Anemia/blood disease	Yes	5	3.6	27	2.6	0.409
	Malignant neoplasm	Yes	13	9.5	119	11.5	0.566

The effect of education and occupation on the frequency of dementia, before and after adjusting for lifestyle variables and medical history, is shown in Table 2. After adjustment for the interaction term of age and educational attainment, higher age and lower educational attainment significantly increased the odds ratio for dementia, whereas the interaction term of sex and educational attainment was not significant. The frequency of dementia increased as SES decreased. Participants with educational attainment of ≤6 years were more likely to have dementia (age- and sex-adjusted OR 3.27 [95% CI: 1.84–5.81]; model 1). The OR for educational attainment of 7–9 years did not differ significantly from the OR for educational attainment of ≥10 years. After the models were also adjusted for occupation, lifestyle factors, and medical history, the OR increased to 3.23–3.56 (models 3–5). Blue-collar workers were also more likely to have dementia (age- and sex-adjusted OR 1.26 [95% CI 0.80–1.98]; model 2); the OR remained stable after additional adjustment for occupation, lifestyle, and medical history (models 3–5). After being adjusted for age, the OR increased for men but not for women (model 1). Furthermore, the OR significantly increased for men after adjustment for lifestyle factors (model 4). Age was strongly associated with dementia risk. Alcohol consumption and smoking were independently associated with dementia. The OR for dementia was higher for former drinkers than for nondrinkers and lower for former smokers than for nonsmokers. The OR for dementia was also independently associated with medical history (i.e., diabetes, Parkinson’s disease, stroke, and angina pectoris/cardiovascular disease). After adjustment for all potential confounders (model 5), low educational attainment, male sex, older age, former consumption of alcohol, diabetes, Parkinson’s disease, stroke, and angina pectoris/cardiovascular disease history were found to increase dementia risk, whereas former smoking was found not to increase the risk.

**Table 2:** Dementia’s association with socioeconomic status before and after adjusting for lifestyle factors and medical history

		Model; odds ratio (95% confidence interval)				
	Crude odds	1	2	3	4	5
Educational attainment						
≤ 6 years	8.85 (5.26–14.87)	3.27 (1.84–5.81)		3.23 (1.79–5.84)	3.45 (1.88–6.34)	3.56 (1.90–6.66)
7–9 years	1.12 (0.74–1.69)	0.92 (0.59–1.43)		0.89 (0.56–1.41)	0.93 (0.58–1.49)	0.92 (0.57–1.50)
≥ 10 years	1.00	1.00		1.00	1.00	1.00
Occupation						
White collar	1.00		1.00	1.00	1.00	1.00
Blue collar	1.61 (1.06–2.46)		1.26 (0.80–1.98)	1.16 (0.72–1.90)	1.12 (0.68–1.84)	1.14 (0.68–1.90)
Both	1.32 (0.76–2.28)		1.33 (0.74–2.39)	1.29 (0.71–2.36)	1.25 (0.67–2.31)	1.07 (0.56–2.02)
Other	2.65 (1.40–4.99)		1.90 (0.94–3.84)	1.96 (0.96–4.01)	1.74 (0.83–3.61)	1.66 (0.78–3.53)
Age						
65–74	1.00	1.00	1.00	1.00	1.00	1.00
75–84	4.55 (2.59–7.99)	4.14 (2.34–7.33)	4.51 (2.56–7.94)	4.08 (2.30–7.25)	3.65 (2.04–6.54)	3.55 (1.94–6.49)
≥ 85	19.03 (10.79–33.59)	14.61 (8.01–26.65)	19.11 (10.72–34.07)	14.21 (7.77–25.99)	12.61 (6.78–23.46)	12.35 (6.41–23.79)
Sex						
Men	0.92 (0.64–1.32)	1.34 (0.90–2.01)	1.31 (0.87–1.96)	1.42 (0.94–2.17)	3.05 (1.60–5.83)	2.71 (1.39–5.27)
Women	1.00	1.00	1.00	1.00	1.00	1.00
Alcohol consumption						
Current drinker	0.41 (0.25–0.67)				0.52 (0.28–0.97)	0.61 (0.32–1.16)
Former drinker	2.94 (1.85–4.69)				2.61 (1.47–4.65)	2.52 (1.39–4.59)
Nondrinker	1.00				1.00	1.00
Smoking						
Current smoker	0.39 (0.17–0.91)				0.40 (0.15–1.09)	0.50 (0.18–1.36)
Former smoker	0.76 (0.50–1.16)				0.38 (0.20–0.73)	0.36 (0.19–0.70)
Nonsmoker	1.00				1.00	1.00
Diabetes						
Yes	1.96 (1.29–2.98)					2.03 (1.23–3.35)
No	1.00					1.00
Parkinson’s disease						
Yes	5.54 (2.07–14.81)					3.84 (1.11–13.37)
vNo	1.00					1.00
Stroke						
Yes	2.93 (1.80–4.76)					2.59 (1.45–4.63)
No	1.00					1.00
Angina pectoris/cardiovascular disease						
Yes	2.64 (1.62–4.31)					1.81 (1.02–3.20)
No	1.00					1.00
Dental disease/oral disease						
Yes	0.55 (0.28–1.07)					0.55 (0.26–1.15)
No	1.00					1.00
Bone fracture						
Yes	2.56 (1.70–3.86)					1.56 (0.95–2.55)
No	1.00					1.00

Model 1 was adjusted for age, sex, and educational attainment

Model 2 was adjusted for age, sex, and occupation

Model 3 was adjusted for age, sex, and SES

Model 4 was adjusted for age, sex, lifestyle, and SES

Model 5 was adjusted for age, sex, lifestyle, medical history, and SES

ORs for lifestyle factors and medical history are shown in Table 3. Neither alcohol consumption nor smoking was related to educational attainment or occupation. The risk of stroke was higher for those who had held both white- and blue-collar jobs than for those who had held white-collar jobs only. Diabetes was seen more in participants with higher levels of educational attainment or white-collar employment.

**Table 3:** Association of socioeconomic status with dementia risk factors

	Alcohol consumption		Smoking		Diabetes		Parkinson’s disease		Stroke		Angina pectoris/cardiovascular disease	
	Yes	OR	Yes	OR	Yes	OR	Yes	OR	Yes	OR	Yes	OR
	%	95% CI	%	95% CI	%	95% CI	%	95% CI	%	95% CI	%	95% CI
Educational attainment												
≤6 years	26.9	0.85 (0.43–1.67)	24.4	1.10 (0.44–2.78)	15.4	0.85 (0.42–1.72)	2.6	1.00 (0.19–5.39)	10.3	1.22 (0.52–2.87)	12.8	0.92 (0.42–2.01)
7–9 years	38.8	1.00 (0.73–1.39)	31.2	1.00 (0.66–1.52)	13.5	0.77 (0.53–1.10)	0.7	0.41 (0.11–1.53)	9.8	1.24 (0.79–1.95)	9.5	1.05 (0.67–1.67)
≥10 years	46.9	1	41.2	1	18	1	1.8	1	7.9	1	8.2	1
*χ*^2^*p*	<0.001		<0.001		0.142		0.229		0.513		0.365	
Occupation												
White collar	40.8	1	35.3	1	17.5	1	1.7	1	6.8	1	7.9	1
Blue collar	46	1.08 (0.76–1.53)	41.1	0.94 (0.61–1.46)	14.3	0.78 (0.53–1.15)	0.9	0.59 (0.17–2.13)	9	1.16 (0.69–1.94)	10.2	1.06 (0.65–1.74)
Both	44.4	1.06 (0.70–1.62)	38.8	1.00 (0.58–1.72)	18.4	1.08 (0.70–1.69)	2	1.36 (0.40–4.65)	12.2	1.79 (1.01–3.15)	8.2	1.00 (0.53–1.86)
Other	30.4	1.63 (0.90–2.97)	11.4	0.62 (0.22–1.74)	12.7	0.78 (0.38–1.60)	1.3	0.72 (0.08–6.08)	10.1	1.61 (0.70–3.71)	11.4	1.37 (0.61–3.05)
*χ*^2^*p*	0.053		<0.001		0.37		0.668		0.147		0.549	

Model: age, sex, educational attainment, and occupations were simultaneously entered

The Sobel test results yielded a contribution degree of diabetes of 0.004% to low SES and dementia.

## Discussion

The present study found that low SES, lifestyle factors, and lifestyle-related diseases were associated with dementia; however, low SES was unrelated to lifestyle factors or lifestyle-related diseases. We found that lifestyle-related diseases such as diabetes minimally acted as a mediator between low SES and dementia.

The association between dementia and low educational attainment was affected by age. Higher age and lower the educational attainment significantly increased the risk of dementia. This is because seniors have a relatively lower educational attainment, and they may be more likely to develop dementia. This study showed that dementia is associated with alcohol consumption, smoking, diabetes, Parkinson’s disease, stroke, and angina pectoris/cardiovascular diseases. Several American and European case-control studies have reported that dementia risk is lower for current drinkers than for nondrinkers [27–29] and lower for current smokers than for nonsmokers [30, 31]. Our results support this. Lifestyle changes, such as alcohol consumption and smoking, significantly increased the risk of male dementia. This was speculated because at the time, these lifestyles were hardly seen in women, and mainly belonged to men. Stroke is the primary cause of vascular dementia, and Parkinson’s disease (Parkinsonism) symptoms are the core component of Lewy body dementia [32]; this may explain how stroke and Parkinson’s disease were associated with dementia in the present study. As for the association between dementia and type 2 diabetes, insulin resistance and hyperinsulinemia are currently thought to be important risk factors for dementia [33]. Hyperinsulinemia may cause accumulation of β-amyloid in the brain. Insulin resistance might also increase β-amyloid. Cardiovascular diseases like angina pectoris are based on arteriosclerosis of blood vessels. Both cerebrovascular and cardiovascular diseases are the results of progressed arteriosclerosis. Arteriosclerosis causes reduction in oxygen and nutrition of the brain because of decreased blood flow and can lead to situations wherein β-amyloid cannot be removed, thereby resulting in Alzheimer’s disease [33]. The results of this study also supported the association between all these diseases and dementia.

In the present study, low SES had a negligible association with key dementia risk factors (i.e., alcohol drinking habits, smoking habits, diabetes, Parkinson’s disease, stroke, and angina pectoris/cardiovascular disease). The role of mediating diabetes associated with low education attainment and dementia was minimal. This indicates that, for low-SES groups in Japan, lifestyle-related diseases (i.e., diabetes) do not necessarily lead to dementia. It has been reported that people in European countries and the United States with low SES tend to have diabetes [34]. On the contrary, the present results show that white-collar workers in Japan tend to have diabetes in higher numbers than blue-collar workers. Similar studies in Japan have reported that low SES is not necessarily correlated with lifestyle-related diseases [35, 36]. In c considered a “disease of affluence” by the 1950s. People with low SES before the 1950s, who correspond to low SES seniors in our survey, underwent physical labor and consumed a grain and vegetable-centered diet. We conclude that the lower incidence of diabetes in the low SES group is because of such a social background in the earlier days.

Dementia risk was approximately three to four times greater for participants with ≤6 years of education than for participants with ≥10 years of education, even when adjustments were made for age, sex, occupation, lifestyle, and medical history. This suggests that low educational attainment is an important dementia risk factor. The education OR remained significant after adjustment for other potential risk factors. The duration of compulsory education in Japan has been extended from 6 to 9 years (aged 6–15 years) since 1947. The number of generations that only receive 6 years of compulsory education will decrease; therefore, the influence of such inequalities in educational attainment will decline in the future. Dementia risk was lower for participants with ≥7 years of education, which illustrates the importance of primary and secondary education.

The results of the present study indicate that limited education alone can be a risk factor for dementia. These results may also lend support to the cognitive reserve hypothesis, whereby the nerve cells of individuals with advanced educational attainment are highly active, which delays the onset of dementia [37]. The cognitive reserve hypothesis also suggests that greater participation in social or leisure activities in later life may protect against dementia [38]. While there are difficulties in increasing educational attainment beyond a certain age, increased awareness of the benefits of active leisure activities and social engagement may still be highly beneficial.

Several study limitations should be noted. First, the amount of alcohol consumed by current and former drinkers was not examined. Second, additional predictive factors such as exercise and activity that could have accounted for the long time interval between the establishment of the SES, educational attainment, employment history, and subsequent dementia of participants were not addressed. Third, responses were collected retrospectively rather than ad hoc, which could have data accuracy implications. For diabetes history, for example, family members might say that their parents did not have diabetes when in fact they did, but the reverse would not happen. Therefore, more subjects with dementia may have diabetes than the results shown in this survey. This tendency will strengthen the association between diabetes and dementia. Fourth, there is a possibility of selection bias, as we may have chosen participants who are relatively more cooperative and lead relatively healthier lifestyles. However, it is unlikely that participants without dementia refused the phase II survey. Compared with the HDS-R high-score group, the HDS-R low-score group had a significant medical history of diabetes. Even assuming that the participants who refused to take the phase II survey had all been diagnosed with dementia would not weaken the association between diabetes and dementia. Fifth, a survival bias is possible because of the study design; we could not collect data from individuals who died by the end of this survey. It has been reported that individuals in high-SES groups live longer than those in low-SES groups [39]. c rate multiplied by the average disease duration, the duration of dementia in high-SES groups is longer than that in low-SES groups. The present study found the prevalence of dementia in low-SES groups to be higher than that in high-SES groups; therefore, the incidence rate of dementia in low SES is higher than that in high SES. This suggests that low SES may strongly affect dementia, even when we consider survival bias.

The present study shows that in Japan, the mediation of low SES and dementia by lifestyle-related diseases is not as strong in recognition of these limitations. However, there is the possibility of another mediator of low SES and dementia that was not evaluated in the present study. Because the domain of social life, for example, was not examined in this study, further research is required.

## Conclusions

The present study found low educational attainment with ≤6 years to be independently associated with dementia. This association was not explained by lifestyle factors or medical history. In Japan, the link between low SES and dementia was found to be minimally mediated by lifestyle-related diseases (e.g., diabetes).

## Abbreviations

CI: Confidence interval
OR: Odds ratio
SES: socioeconomic status
